# Towards Privacy-Preserved Aging in Place: A Systematic Review

**DOI:** 10.3390/s21093082

**Published:** 2021-04-28

**Authors:** Munkhjargal Gochoo, Fady Alnajjar, Tan-Hsu Tan, Sumayya Khalid

**Affiliations:** 1 Department of Computer Science & Software Engineering, College of Information Technology, United Arab Emirates University, Al Ain 15551, United Arab Emirates; fady.alnajjar@uaeu.ac.ae (F.A.); sumayya.khalid@uaeu.ac.ae (S.K.); 2 Department of Electrical Engineering, National Taipei University of Technology, Taipei 106, Taiwan; thtan@ntut.edu.tw; 3 Intelligent Behavior Control Unit, RIKEN Center for Brain Science (CBS), Wako 463-0003, Japan

**Keywords:** elderly, independent living, privacy-preserving, smart homes, sensors, unobtrusive

## Abstract

Owing to progressive population aging, elderly people (aged 65 and above) face challenges in carrying out activities of daily living, while placement of the elderly in a care facility is expensive and mentally taxing for them. Thus, there is a need to develop their own homes into smart homes using new technologies. However, this raises concerns of privacy and data security for users since it can be handled remotely. Hence, with advancing technologies it is important to overcome this challenge using privacy-preserving and non-intrusive models. For this review, 235 articles were scanned from databases, out of which 31 articles pertaining to in-home technologies that assist the elderly in living independently were shortlisted for inclusion. They described the adoption of various methodologies like different sensor-based mechanisms, wearables, camera-based techniques, robots, and machine learning strategies to provide a safe and comfortable environment to the elderly. Recent innovations have rendered these technologies more unobtrusive and privacy-preserving with increasing use of environmental sensors and less use of cameras and other devices that may compromise the privacy of individuals. There is a need to develop a comprehensive system for smart homes which ensures patient safety, privacy, and data security; in addition, robots should be integrated with the existing sensor-based platforms to assist in carrying out daily activities and therapies as required.

## 1. Introduction

Progressive population aging is a global phenomenon. Improvements in public health, medicine, nutrition, and workplace safety standards have contributed to higher life expectancy. According to a United Nations report, the population aged ≥ 65 years is projected to be approximately 2 billion by 2050 [1]. This exponential increase in the aging population is liable to impose a significant burden on the socioeconomic well-being of many countries. Healthcare systems across the world will face the challenge of delivering efficient services to better educated, elderly population within strict budgetary constraints.

Elderly individuals tend to face difficulties in carrying out routine daily activities [2], which may make them dependent on caregivers or family members. In addition to the increased prevalence of comorbid conditions, elderly individuals tend to develop cognitive impairment with progression of age. Low physical strength [3], age-related dementia [4], depression [5], behavioral changes [6], and compromised communication skills [7] are some of the other issues that contribute to the increased dependency of elderly people.

Placement of the elderly in a nursing home or a care facility against their will has a detrimental effect on their well-being; it often leads to social isolation, depression, and greater dependency for completing self-care tasks [8]. Elderly people typically prefer to live in their homes rather than in a facility even when they require specialized care. In a survey, 30% of individuals over the age of 65 years were of the opinion that they would “rather die” than enter a nursing home [8]. The considerable costs involved in the care of an aging population and the perennial shortage of healthcare professionals [9] have prompted efforts by both industry and researchers to develop and test alternate models of care [10]. In particular, several studies have investigated the feasibility and effectiveness of systems for assistance and health monitoring of elderly in their home environment [11].

Remote health monitoring by leveraging technology is an efficient and cost-effective solution [12] to promote the well-being of the elderly by allowing them to age in place within the comfort of their own home; at the same time, they can be monitored and taken care of with use of technology that can predict any abnormal behavior and can alert the caregivers accordingly. This technology can allow an elderly person to live independently without the constant need for help from a family member or friend, boosting their moral and confidence. These remote monitoring systems do not intrude the daily lives and also allow real-time tracking of important functional parameters with few human resources and at reduced cost. The main objective of all smart solutions is to improve the quality of life (QoL) of the user [13]. The World Health Organization (WHO) defines QoL as the “*individual’s perception of their position in life in the context of the culture and value systems and in relation to their goals, expectations, standards and concerns. It is a broad ranging concept affected in a complex way by the persons’ physical health, psychological state, level of independence, social relationships and their relationship to salient features of their environment*” [14].

Artificial intelligence (AI), and machine learning algorithms are being widely used in smart technology applications. They may be applied in the entire network of sensors or just may be to as a part of the smart home. It can help in device management, energy conservation, health monitoring, intelligent communication, security and assistive personal robots by using data processing, prediction-making, voice recognition, decision-making and activity recognition [15]. Voice and image recognition via AI are being used in many smart home products, whereas prediction-making, activity recognition and data processing are being widely studied and enhanced.

Development of an effective and impactful smart home requires investigation of various susceptibility issues associated with the elderly people. These homes offer a safe and welcome space for elderly. Safety is the top priority for these concepts of smart care [16]. But there are chances of emergency events or incidents, which may occur due to the old age nature, like falls or urgent health issues. However, the current technology has incorporated methods to prevent and detect critical situations [17]. The advancement of technology allows the applied models to detect if a particular situation or event is an emergency or not. These methods can be used and provide immediate assistance when required.

Preserving the user privacy is another crucial aspect that needs due attention in smart home development technology. In Internet of Things (IoT) applications, users provide personal data to different devices and services, whereby privacy becomes extremely vulnerable [18,19,20].

In particular, location-based service (LBS), which uses personal data of users, is becoming increasingly popular [21]. Preservation of users’ identity (and hence the personal information) from the service providers (SPs) has attracted the attention of many researchers. The technology needs to be robust to protect the privacy and be non-intrusive, so that the users can trust their systems and take full advantage of it. Existing architecture needs to be more inclined towards security and privacy [22]. A less secure smart home can lead to exposure of the personal information of users to strangers or malicious entities. To the best of our knowledge, no standard definition for privacy-preserved age in place is available in the literature.

The usage of various technologies like sensors, cameras, robots, artificial intelligence, voice-based protocols, in a home or a building may improve the quality of life, well-being, energy conservation, safety and productivity [23,24,25,26,27]. The utilization of Internet of Things (IoT) has increased, creating a network of different objects, like sensors, actuators, mobile devices, tablets, etc. [28].

These remote monitoring technologies use sensors [29,30,31,32] and cameras [33] to monitor the elderly, detect falls or for emergency situations. Developments in technology have led to usage of wireless sensor networks placed all over the house at designated points, which collects the data and is usually processed and analyzed by controlled thru a suitable algorithm to give report to the caretaker or healthcare provider. Various types of sensors, have been used, be it environmental sensors, water sensors, temperature sensors, wearable sensors to keep track to numerous activities like, water usage, sleeping patterns, walking patterns, eating patterns, etc.

Several reviews have assessed the requirements of older adults in the context of home-based health care or telecare [34,35]; however, two systematic reviews specifically examined the facets of aging in place, i.e., a systematic review on cost effectiveness of aging in place [36] and a systematic review of acceptance of technology for aging in place [37]. The authors of the first review [36] found that the existing technologies were of low quality; in addition, the authors were not able to draw any definitive conclusions owing to lack of standardization of measurement indices in various studies. The technology acceptance review identified issues pertaining to discretion and affordability, control and freedom, and anxiety of stigmatization and institutionalization [37]. These two reviews have contributed significantly to the understanding of the concept of aging-in-place; however, each review was focused on a single aspect of the lived experience. Another review [38] synthesized and evaluated the existing qualitative evidence pertaining to aging in place in the US. The value of aging in place is determined and affected by various factors such as culture and differences in economic and social structure [39]. This review [38] was based on experiences in the US and the findings may not be entirely generalizable to other countries.

Few reviews talk about the application of AI technology to smart homes. One review [40] evaluated the intelligent surveillance systems in smart home environments, another review [41] investigated philosophical keystones and how they assist healthcare workers, scientist to collaborate with engineers to develop intelligent health-assistive smart homes. Kumar et al. [42] discussed the different variety of home automation systems and how they use AI tools. These systems were mostly applied as comfort ability, remote control, optimal resource utilization, and security.

It was noted that there were very few reviews which target the protection of privacy in the smart homes designed for the elderly. Given the availability of literature there is a need to review the current strategies and how they can be further enhanced. The current status of the research needs to be analyzed and evaluated for the various features that constitute in development of smart home, how each of these features can be further advanced in terms of technology and usability, how they can be made secure and unobtrusive, so that the users do not feel that they are under surveillance. The objective of this systematic literature review was to identify the different types of smart-home solutions or technology-based strategies available to assist the elderly to live independently in their homes and assess the current state of privacy preserving technologies incorporated into these homes.

## 2. Methods

A systematic literature review (SLR) condenses existing proof, identifying holes and identifies directions for future research.

### Search Strategy

An extensive literature research was performed in the PubMed, SCOPUS and IEEE Xplorer databases. The keywords used were “smart homes”, “elderly”, “aging in place”, “non-intrusive”, “privacy preserving”, and “independent living”. They were used in combinations like the following:

- Smart Home AND (Elderly OR Aging in place) OR (Non-Intrusive OR Privacy)
- Smart Home AND (Elderly OR Aging in place) AND Non-Intrusive
- Smart Home AND Elderly AND Privacy

Using advanced search techniques, the databases were searched, in combinations of these terms appearing in all metadata (title, abstract, keywords, full text) and were later screen according to various criteria mentioned in the next section.

The retrieved articles were shortlisted, and duplicate publications were removed. Subsequently, the titles and abstracts of the remaining studies were screened against the following criteria.

- The inclusion criteria were:
- Studies published in English.
- Studies that used technology in the home, both technologies embedded in the home or independent technology (such as a robot).
- Addressed the needs of older adults living independently both healthy and elderly with health issues (monitoring of activities of daily living or health).
- Studies that entailed implementation or deployment of technology, even if in a pilot form, or proposed studies, to assess the feasibility and outcomes.
- Studies that were published within the last decade, so that the latest researched were included.

The exclusion criteria were:

- Studies published as academic theses.
- Studies which were reviews, book chapters.
- Studies which were not health-related and focused on other aspects such as energy-conservation or security surveillance systems

**Figure 1:**
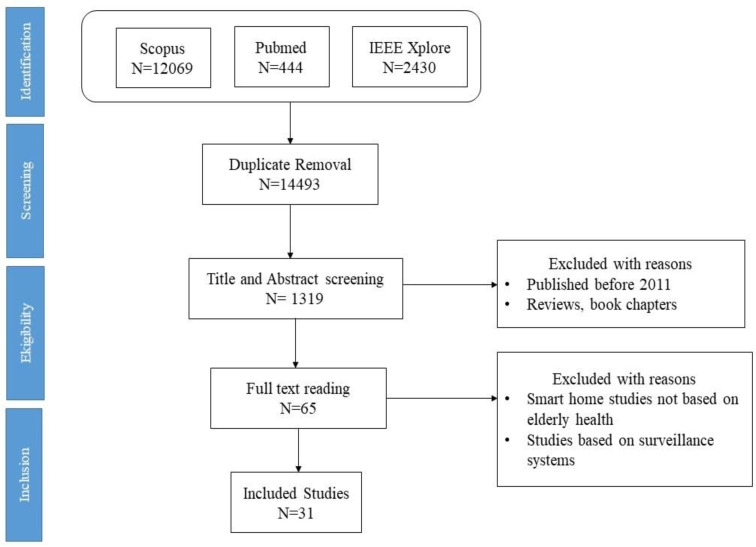
Schematic illustration of the literature search.

Figure 1, shows the selection strategy using PRISMA flowchart.

## 3. Results

A total of 1319 studies were identified after title and abstract screening, out of which 65 were accessed for full text reading and as a result 31 were finally included in the review. The features available for smart home systems that were found in the shortlisted studies can broadly categorized into the following categories:

### 3.1. Application of Environmental Sensors, Wearables and Cameras

Twenty-nine studies [43,44,45,46,47,48,49,50,51,52,53,54,55,56,57,58,59,60,61,62,63,64,65,66,67,68,69,70,71] included in this review entailed the use of various types of sensors; mostly environmental sensors, as the key elements involved in the functioning of a smart home. These studies entailed deployment of entire sensor-based network systems or placement of various sensors all over the home for monitoring the ADL and the overall well-being of subjects.

### 3.2. Security and Privacy of Data

Eleven studies [43,44,47,49,51,55,58,59,60,65,67] have been more focused in developing smart homes with protection of user privacy being the utmost priority. 

### 3.3. AI Machine Learning and Robots in Smart Homes

Twenty-seven [43,44,45,46,47,48,49,50,51,52,53,54,55,56,57,58,59,60,61,62,63,65,67,72,73] out of the 31 studies used AI, machine learning, or robots in their smart home techniques.

### 3.4. Usage Safety, Emergency Services and Fall Detection

Four studies [45,60,63,71] out of 31 studies had incorporated some linkage with emergency services. Nine studies [44,45,46,53,58,60,61,66,67] out of 31 studies investigated technologies with in-built fall detection capability

### 3.5. User Feedback, Satisfaction and Effects of Smart Homes

Eight [43,44,58,60,63,66] of the 31 studies included in this review, the systems deployed were aimed at providing some sort of medical support by health monitoring and taking appropriate action. Fourteen [43,45,47,48,49,51,52,53,55,60,64,65,69,72] of the 31 studies were aimed at monitoring the environment for any abnormalities and detecting falls, which allowed the elderly to stay alone in their homes.

**Figure 2:**
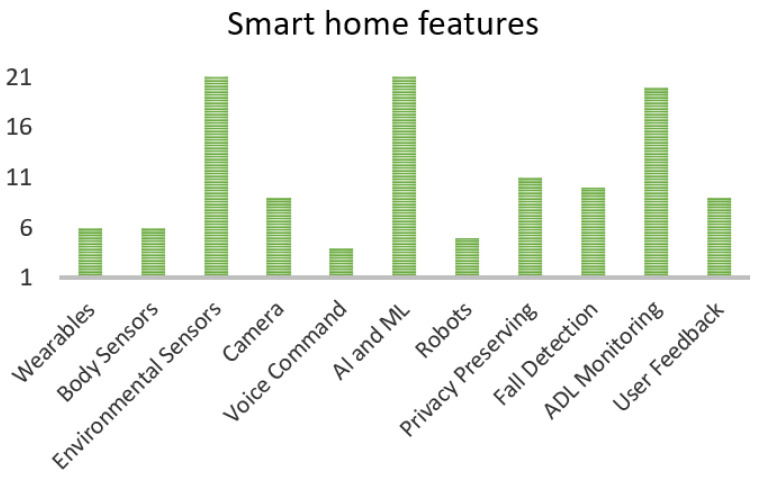
Smart home features in selected studies.

Figure 2 shows the numbers of studies with different features of smart home. This figure enlists in detail features like wearable, body sensors, environmental sensors, cameras, voice command feature, Al or ML capabilities, robots, privacy preservation, fall detection and monitoring of daily activities. Each bar represents the number of studies out of 31 which have included the mentioned feature.

Table 1 below summarizes the key characteristics of these studies. Table 2 enlists the different features and tools used in the shortlisted studies, such as wearable devices, environmental sensors, cameras, robots, voice commands; in addition, we assessed whether these systems had safeguards to protect user privacy.

## 4. Discussion

This systematic review was conducted to showcase the range of currently available smart-home technologies that improve the quality of life of the elderly, while maintaining their privacy and comfort. Thirty-one studies conducted during the period 2010–2020 were included in the review. The topic of smart homes is very broad and can be looked at through many different perspectives: security [74], safety [75], health monitoring [76], social interaction [77], general well-being [43], support for carrying out activities of daily living, timely reminders for certain tasks or intake of medications. This review mainly focused on the aspects of health monitoring and environmental monitoring with use of technology involving the use of sensors, wearables, and robots. In addition, we assessed any potential concerns pertaining to the privacy of users.

### 4.1. Application of Environmental Sensors, Wearables, and Cameras

The smart-home technologies are now becoming increasingly non-intrusive as more people are becoming aware of the dangers of privacy breach; at the same time, these entail continuous monitoring of the well-being of the user. Most of the technologies are for monitoring the health of elderly people through use of sensor-based platforms, wearable devices, robots, or by simply monitoring the environment to notice any unusual activity or anomaly. These systems generate reports or inform the caregiver in case of any anomaly.

Kim et al. [43] used sensors to monitor the mental health of the subject; these sensors helped detect normal behavior or mild depression with 90% accuracy. Deen [44] used sensing technology to monitor the walking patterns, which helped in early detection of muscle weakness, fall, and improper sleeping patterns. Lotfi et al. [55] employed wireless sensors and a computerized base station to monitor patients with dementia who were living independently. The information from the sensors was transmitted to care givers through a centralized portal and helped predict any anomalies. Do et al. [58] used a system which included home-based robot, sensor network, body-based sensor network, a mobile device, cloud-based servers, and remotely accessible caregivers. Grguric et al. [65] developed a sensor-based system with AI capability to learn the subject’s behavior over time and detect any abnormal behavior. Yu et al. [66] medication adherence in the subjects. Tsukiyama [63] used water-flow sensors, IR-based motion sensors and radio-frequency identification (RFID) receivers to screen the daily activities of an elderly and sense any atypical conditions. The water flow sensors monitored the usage of water during urination, kitchen activities, and self-hygiene to maintain a check on any deviation from a healthy lifestyle.

Environments and platforms using several types of sensors (such as motion sensors, water sensors, light sensors placed at designated spots in various parts of the home) help in carrying out ADL, fall detection, and other activities. The usage of camera and wearables was very limited. In the 31 studies reviewed, only five studies [46,66,67,68,71] entailed the use of a camera. Wilson et al. [46] used to monitor the elderly with their permission. Portet et al. [67] used camera only for social communication between the elderly and their families and friends. Yu et al. [66] used a fall detection system based on computer vision, while. Hattink et al. [68] used camera as a part of their surveillance system to monitor falls and emergencies. Hu et al. [71] used a camera array to detect falls and alert the caregivers.

Wearables do not pose any threat to privacy, however, it is not feasible or practical to constantly wear one. Moreover, wearables typically have a relatively short battery life, require maintenance, and cause discomfort over long usage if required to wear consistently throughout the day., may lead to allergic reactions, rashes [78]. Six studies [44,50,58,59,61,71] out of 31 used wearables in the form of body sensors, smartwatch, and monitors. Kshirsagar et al. [50] proposed a wearable glove-based system, with embedded flexural sensors, microcontrollers and Bluetooth features. The gesture generated signals which controlled various home appliances through a mobile application. Jekel et al. [52] included subjects with MCI along with healthy older adults, they carried out their study by setting up a two-room furnished flat with sensors on the items in the house and the subjects were asked to carry out simple tasks, sensor data was monitored and helped in the prediction of MCI or deteriorating cognitive functions. Hattink et al. [68] developed a system called Rosetta for their MCI and dementia diagnosed subjects. The system consisted of three subsystems, one supported in carrying out all the daily activities, another system recorded the data for analysis, third system detected any anomalies, emergency situation like fall or medical assistance. However, certain challenges have not been addressed. These include use of sensors in the washroom or during bathing, sensors to detect falls, and sensors which can distinguish between the elderly and their pets (animal companions to cater to the loneliness) or visitors and residents.

Rizvi et al. [54] developed an Android-based system, comprising of two modules—a GSM module and a Bluetooth module—which allowed users to control the home devices both remotely and locally through custom designed mobile application. The targeted subjects were elderly and handicapped people. Nisar et al. [56] also developed an android based smart home system where the application had three modules: the sensor module, the controller module and actuator module. Sensor-based devices could be accessed through smartphones both remotely and locally, thus making life efficient for the elderly while also reducing power consumption.

### 4.2. Security and Privacy of Data

Security and privacy are key concerns when designing a smart home. Users are generally apprehensive about their privacy as well as data security [79]. Proper ethical agreement must be obtained prior to the use of any video or IP cameras for observation purpose to allay these concerns. Since the end devices frequently transmit data to a central controller, simple eavesdropping attacks can lead to data leaks; the types of end devices can expose the identity of the user. Thus, potential attackers can infer when the house is vacant or identify who is present in the house so that they can break in or cause severe situations. However, none of the 31 studies had explicitly mentioned any such data security feature in their technologies.

A trend was discernible in the 31 studies, wherein studies carried out in recent years accorded due attention to make the technology as non-intrusive and privacy-preserving as possible. Security and privacy are major areas of concern as duly pointed out in several studies [80,81,82]. They have used non-intrusive techniques for achieving their target outcome with the help of different kinds of sensors, wearables and robots. We have developed several deep learning models [83,84,85,86,87] based on privacy-preserved activity and posture recognition tasks; however, three [85,86,87] of these studies were for eldercare ADL monitoring, these studies are not included in this review paper as they have employed an open dataset. Also, most of these techniques do not employ cameras in order to make the users more comfortable. Less usage of cameras and wearables is an added advantage.

### 4.3. AI Machine Learning and Robots In Smart Homes

AI and machine learning are two remarkable innovations that can help in the development of highly advanced and smart strategies. Utilization of AI, machine learning, and fuzzy logic can render the systems more efficient and help them produce more reliable and accurate results. Cutting-edge sensing techniques and machine learning strategies are being used in smart homes to autonomously respond to the needs of their users; however, they are rooted in the environment.

The RiSH [58] comprises a robot for home service, a sensor network deployed across the home, a sensor network for monitoring body activities, a mobile device, cloud-based servers, and remotely available caregivers. The robot embedded in RISH had the capability to recognize 37 distinct individual activities through sound actions and was able to identify falling sounds with 80% accuracy at the frame level. The study demonstrated the ability of RiSH and the home service robot in observing and supporting the resident. Grguric et al. [65] used artificial intelligence theories of decision making, reasoning, and pattern recognition based on the advances in ambient intelligence (AmI), sensor networks, and human-computer interaction (HCI). The system studies a person’s behavior patterns without invading their privacy and signals the caregiver(s) in case of detection of an abnormal situation. Iakovakis et al. [61] used a fuzzy logic-based assistive tool for prevention of falls in patients with Parkinson’s disease. The system gathered important signal information from smartwatch and other home-based motion sensors to monitor the risk of fall due to orthostatic hypotension. Rudzicz et al. [70] investigated the use of a mobile robot designed to assist in ADL of elderly people with Alzheimer’s disease by monitoring visuals and providing verbal prompts in difficult situations. Fischinger et al. [73] used a robot called ‘Hobbit’ that assisted the elderly living independently at home. The robotic system adequately performed its core tasks and the subjects were able to perform all tasks with support of the robot. Wilson et al. [46] developed a robot activity support system (RAS) comprised of a sensing network that interacted with the robot; the system detected activity errors in the everyday environment and provided appropriate assistance. For example, it provided physical assistance by locating the key objects required for ADL in the home. Dawadi et al. [48] developed a network of motion and temperature sensors, which scrutinized the daily activities; a machine learning algorithm processed the collected data to compute the task quality, task accuracy, and task sequencing scores. Bianchi et al. [72] proposed a wearable device integrated with deep learning techniques, which recognized most common daily living activities. Saunder et al. [57] used a commercially available robot for their study. They described the teaching and learning method, where the robot is first taught about all the requirements and logistics of a sensor-based house, and once the robot learns it can assist the subjects in their daily living activities. Due to this methodology the robot can easily be customized to meet individual needs, the subjects found this method to be very easy to use and helpful.

### 4.4. Usage Safety, Emergency Services and Fall Detection

Ample importance has been accorded to user safety during the development of smart technologies for home, especially those for monitoring the health of elderly patients with dementia, Alzheimer’s, or Parkinson’s disease. These patients are more comfortable in their own homes owing to their familiarity with the environment; use of these smart technologies can inculcate a sense of security and alleviate fear and anxiety. These technologies empower these elderly people to recollect their daily tasks (e.g., taking medicine, drinking water, etc.), make them more self-sufficient, reduce their social isolation, and enhance their sense of self-worth. Some studies investigated the use of systems that periodically send reports to caregivers pertaining to the activities carried out by elderly and also notifies in case of any anomaly; however, very few studies have emergency services embedded into the system.

Taramasco et al. [60] embedded an emergency button which can place a call to the caregivers and is also connected to emergency and fire-fighting departments. Tsukiyama [63] deployed a system that assesses the health condition of the elderly and forecasts any emergency situation to a local healthcare center without any explicit user interaction. Fischinger et al. [73] employed a robot which can detect emergency and handle the situation appropriately. Fall detection technology is an essential element of any smart home technology for elderly. Elderly people are more vulnerable to falls due to age-related conditions such as muscle weakness, arthritis, and muscle atrophy. Falls may lead to severe injuries that necessitate medical help.

Deen et al. [44] employed a system which can detect health issues, muscle weakness, and fall through a smart walking monitor and smart joint monitor. Portet et al. [68] used a system with the ability to detect fall and help subjects in calling for help. Do et al. [58] used a robot which was able to detect fall sounds with 80% accuracy. Taramasco et al. [60] incorporated special falling sensors in its tele monitoring ADL platform to detect falls. Iakovakis et al. [61] used a fuzzy logic based assistive tool for fall prevention. Yu et al. [66] used a computer vision-based fall detection system for monitoring an elderly person in home care. Fischinger et al. [73] used a care robot which was able to prevent and detect falls. Gnanavel et al. [53] also include a fall detection system, including a heartbeat sensor, pressure sensor and temperature sensor and alerted the caregivers via SMS in case of any anomaly. Hattink et al. [68] had a surveillance system, which was able to detect inactivity, and was able to predict and alert the caregivers of an emergency situation. Hu et al. [71] was a camera P2P based system which detected falls and alerted the caregivers.

### 4.5. User Feedback, Satisfaction and Effects of Smart Homes

The response of the people towards usage of these smart-systems also seems affirmative [50,51,52,57,71,72]. A study [62] showed improved medication adherence among subjects with use of used water sensors to monitor the usage of water to check maintenance of a healthy lifestyle. However, the positive effects cannot be generalized as these studies were performed with small sample sizes of less than 50; moreover, proxy subjects were used in some cases [58].

A trend observed in the 31 studies was that the deployed smart systems were able to achieve their targeted outcome; moreover, the users rated the systems as sensors to track the medicine intake. Others studied usability [65] and acceptability [88]. However, each of the studies had some limitations and none of the studies replicated a model of a complete smart home. The study by Do et al. [58] used a system that is closest to a complete smart home solution; it includes a home service robot, a home sensor network, a body sensor network, a mobile device, cloud servers, and remote caregivers. The system monitors the ADL, informs the caregivers in case of any anomaly, has a robot at hand to assist in ADL with the ability to recognize 37 daily activities and detect falls. The only limitation was that their technology required the use of a wearable device; as discussed above, wearables are not very comfortable for constant daily use. In addition, there was no fall detection technology used in the bathroom, which is a very high-risk area for falls.

#### 4.5.1. Statistical Analysis

If we observe quantitatively 80% of the studies used some form of sensor embedded in their systems, be they environmental sensors, body sensors, motions sensors, etc. These sensors are the crux of a smart home system as they can monitor and record every move, without hampering inhabitants’ daily life, invading their privacy, and also through these sensors, the daily activities can be made efficient and easy to be carried out by subjects with minimum effort. Nearly a fifth (19.3%) of studies used wearables, it was seen that incorporation of wearables in the studies has decreased with time, in recent years, studies are now more focused on sensors and robots to enable a good functional smart home for elderly. Over a third (35.4%) of studies declared usage unobtrusive or privacy-preserving techniques in their methodology, few didn’t mention privacy or their unobtrusiveness, even though this is a very low percentage considering this is an important feature, more focus needs to be put in to include privacy and security feature of a smart home, as with advancing technology, there are new ways to breach security and theft of data can be dangerous. Moreover, it puts the subject at ease if they know that the system is secure and protected and their personal data is safe. Nearly 90% (87.09%) of studies have incorporated AI, ML or robots in their smart home research, as these strategies are proving to be more efficient and beneficial, though they are still in their infancy, more research should be put in to incorporate more features and make it more easy to use for the elderly 

The studies were reviewed were RCTs, pilot studies, experimental studies and also proposed studies. Nearly a fifth (19.3%) were proposed studies, which catered to different subjects like MCI, handicapped and dementia patients or healthy elderly. These studies show a great potential in their research but needs to be validated by including subjects and conducting trials to verify their claim. Few studies open a new window in regard to care of MCI and dementia diagnosed elderly via a smart home strategy. These studies have potential to benefit them immensely in their daily activities, help them live independently, while keeping the caregiver at ease, with emergency and fall detection alert techniques.

To summarize the main features of the smart home technology in the included literature: 80% of the smart home systems reviewed used sensor-based platforms, 29.1% used cameras, 19.3% used wearables, 35.4% used unobtrusive methods, 16.1% used robots, 70.9% used AI and machine learning and 32.2% had fall detection capabilities.

#### 4.5.2. Recommendations for Future Research

There is an inevitable compromise between utility and feasibility. Multiple hardwired installation may be needed at all positions where support could be required (e.g., bathroom, kitchen, and bedroom), which may not be very cost effective. Conversely, installation of too few units may introduce gaps where activity will not be noticed. Integration of robots with smart homes can help with some of these tradeoffs. Moreover, a physically embodied entity like a robot will have greater chances of acceptance than an embedded system [88,89].

Also, robots along with the sensor technology could be a better strategy, as robots can be programmed to assist in ADL (bringing the medicine, reminding of tasks, keeping company, aiding in physical needs like sitting as standing [90], calling the caregivers, etc.), while sensors can be used to monitor the environment. Moreover, there are other determinants of QoL apart from health. Most of the studies employed systems aimed at providing some sort of healthcare.

Satisfaction with and acceptability of any smart home systems is culture-dependent and thus varies in different societies. Age and gender seem to influence people’s idea of space [88], which can also affect the acceptability of a system, in particular where behavior is continuously monitored. Identification of the level of user acceptance is a major challenge for system developers [91].

However, as seen the technology readiness for these systems was rated as low. These systems need to incorporate more mechanisms to protect user privacy and data security in order to gain the trust of their users. Apart from monitoring the health status, these systems should incorporate means of entertainment and companionship to ward off loneliness and anxiety. None of the 31 included studies can be considered a complete smart home system, which is unobtrusive, monitors health, has emergency features, helps in ADL together with keeping them motivated and less lonely and less anxious. These measures would provide a happy and positive place to age in place. In addition, more robust safety features should be incorporated in such technologies to achieve a complete smart home solution. Inclusion of entertainment and gaming [91], social companionship [92], and constant support and assurance can help improve the usability and acceptability of the systems, especially among the elderly, especially for those who live independently, as they will be able to keep themselves busy through these features and not feel depressed.

There is a paucity of research on this aspect. None of the 31 studies reviewed had incorporated entertainment as an add-on option in their system, although one study [72] did have the option of video conferencing with friends and family members. Four studies [48,59,71,72] included robots which could provide some sense of companionship to the user. Future research should focus on these aspects along with monitoring the activities and providing healthcare linkage.

To summarize the main features of the smart home technology, out of 31 chosen literature references 27 used environmental sensors, 22 studies used AI or ML techniques in their strategies, 20 systems could monitor activities of daily living, 11 systems used privacy-preserving methods, 10 used fall detection techniques, 9 studies collected feedback from the user, 9 used cameras in their systems, 6 used wearables, 6 used body sensors, 5 involved robot-based methods and 4 used voice commands. Figure 2 depicts this f information in the form of bar graph.

To conclude, a complete smart home should include a strategically designed sensor-based platform which can function with multiple residents in the house; in addition, non-intrusive fall detection sensors should be installed in washrooms. Emergency buttons should be easily accessible to provide ready access to emergency services. Use of cameras and wearables should be minimized. Integration of robot with the system can assist in ADL, provide medication reminders, and inculcate a sense of companionship [83] to alleviate depression and anxiety. Data security and privacy should be accorded highest priority in the development of smart home solutions.

## 5. Conclusions

Use of smart-home technology for improving the QoL of older adults has received a generally positive response. The studies included in this review for the most part achieved their target outcomes. However, 50% of the studies pertained to monitoring the ADLs of the subjects and informing their caregivers in case of any abnormalities or discrepancies; the other studies deployed systems to achieve very specific tasks such as checking the medication adherence, monitoring the water flow, and analyzing the walking and sleep patterns.

While most of the studies achieved their objectives, none of the studies can claim to have achieved the objective of implementing a complete smart home. Future studies should incorporate all the key features required in a smart home: individual privacy, monitoring via sensor-based technology, assistance in daily activities via a home robot, provision for connecting to caregivers, access to emergency assistance, and predicting depression.

## Figures and Tables

**Table 1:** Summary of the studies included in the review.

No.	Reference	Subjects	Method Used	Features	Results
1	Kim et al. 2017 [43]	20 Elderly with depression	Privacy-preserved elderly monitoring and mental health measurement system employing unobtrusive environmental sensors and feature learning method Method: Neural network, C4.5 decision tree, Bayesian network and SVM.	Capability detection with accuracy Non-intrusive system Easy installation Long-term monitoring of depression No invasion of personal privacy Cost effective for most elderly	96% accuracy in detecting normal condition as well as mild depression.
2	Deen 2015 [44]	Healthy Elderly Sample size: Not mentioned	Cost-effective smart-home sensing technology consisting of smart sleeping setting, smart monitoring of human joints and walk. Usage of body-based sensors, motion sensors, accelerometers, and gyroscopes for data collection and analysis Method: K Cluster Algorithms	Smart walking monitor to analyzes the walking pattern and features. Smart joint monitor, to monitor the range of motion, flexibility, and balance. The sleeping setting conditions can be adjusted based on the individual’s needs.	Health problems, muscle weakness, fall detection, improper sleeping patterns can be detected at an early stage. Safety and comfort in familiar surroundings, and reduced healthcare cost.
3	Sprint et al. 2016 [45]	3 females aged ≥ 80 years	Smart homes fitted with ceilings having motion/light sensors, doors and temperature sensors, which unobtrusively and continuously monitor the daily activities. Data collected from these sensors were stored in the database, and used to train the CASAS-AR. Method: naïve Bayes classifiers and decision trees, complex models Gaussian mixture models and conditional random fields.	The 3 subjects had different behavior patterns and health events: ◦ Subject 1 underwent radiotherapy for cancer treatment ◦ Subject 2 had insomnia issues ◦ Subject 3 fell and injured her leg Collection and labeling of sensor data was done using activity recognition and BCD was applied to analyze behavioral changes. Analysis of changes in the timing and duration of activities was also done.	Evaluation indicated that the behavioral changes observed in these cases were in line with the medical literature and that the variations can be automatically spotted using Behavior Change Detection (BCD).
4	Wilson et al. 2019 [46]	26 subjects	Robot integration into smart homes of individuals with functional limitations was done to provide support. Robot Activity Support (RAS) system in combination with smart environmental sensors; detection and mapping of objects, interaction with robot, detection of errors in activity and provision of assistance in routine environment. 5Method: single shot multibox detectors (SSD), regions with convolutional neural networks (R-CNN), and region-based fully convolutional network (R-FCN)	RAS robot coached users on steps needed to carry out the activity so that activities of daily living can be performed accurately and completely. RAS: ◦ Sensor-driven recognition of activity errors and the need for assistance. ◦ Searches and locates the resident, provides video reminders, and guides them to the needed objects for the missed activity steps. Physical assistance is provided by showing residents the location of key objects in the home that are required for daily deeds.	Satisfactory impressions of the RAS tablet interface. Neutral and highly variable rating of system usability. Questionnaire ratings were not related to age or comfort with system. The full script video was found to be puzzling and not very helpful to someone with MCI as compared to the “next step” video and object guidance.
5	Alberdi et al. 2018 [47]	29 older adults	Unobtrusive collection of behavioral data of elderly living alone in Smart-Homes. Method: Linear Regression (LinearR), SVr with a Radial Basis Function (RBF) kernel and k nearest neighbors (kNN) algorithms	Sensor events of residence were collected. The AR activity recognition algorithm allocated a particular activity to each sensor entry. The system computed the daily sleep and movement patterns, 17 behavior patterns, time utilized in some specific ADLs, and the overall characteristics of the daily routine. CAAB algorithm was applied to the data to obtain the behavioral figures of each assessment period.	Activity-aware smart home data can foretell all mobility, depression, and cognition/memory symptoms as well as a consistent variation in movement and visuospatial skills related to cognition. Equal contribution was done by behavioral features in the prediction of every symptom.
6	Dawadi et al. 2013 [48]	179 participants	Ceiling was fixed with motion sensors; door sensors were installed on cabinets and doors; selected kitchen items were installed with item sensors. Each room was fitted with temperature sensors, sensors were fitted to monitor water and burner usage, and a power meter was installed to measure the electricity consumption A researcher kept monitoring upstairs though a web camera and using a microphone and a speaker connected remotely to the participant Method: SVM and other ML algorithms,	The sensor events generated by the activities of the participants were stored. 4 fields were used (date, time, sensor identifier, and sensor message) to record each sensor event. Interpretation of the sensor data files and the corresponding video was done by experts Based on the collected data, the machine learning algorithm quantified the quality of tasks Relation between automated feature set centered on smart home sensor data and the direct surveillance scores were identified using machine learning techniques.	Statistically significant correlation was observed between smart home capabilities and task accuracy scores. Valuable information to assess the quality of ADL was derived and implemented in home with improved sensor technology and algorithm design.
7	Aramendi et al. 2018 [49]	29 elderly	Functional health decline was detected automatically by activity-aware smart home; behavioral data was collected using unobtrusive sensors. Method: SVM and algorithm based of SMOTE-based datasets	Passive infra-red (PIR) presence sensors were used to track movement and activities by triggering data streams of sensors. Based on the collected data, regression models were created to calculate absolute and consistent functional health scores. Classification models were able to reliably detect the complete change and positive and negative variations. 8Assessment of functional health was performed every six months using the Instrumental Activities of Daily Living-Compensation (IADL-C) scale	By way of data from activity-aware smart home, as well as a consistent change in these scores, prediction of Total IADL-C score and sub scores can be done Detection of positive and negative fluxes in everyday functioning is difficult using in-home behavioral data; however, alterations in social skills were predictable
8	Kshirsagar et al. 2020 [50]	Proposed study, No subjects	A glove based, gesture controlled smart home to be used by elderly or disabled. Method: artificial neural network and mobile application	Glove connected to electrical devices in the house Glove has flex sensors, microcontroller and Bluetooth module embedded in it. Generated data from each house is secured on a server. A mobile application reads and writes data from database for user. This is used by both subject and caretaker.	The proposed system is successful in helping the especially abled people and elderly. It is able to accurately identify gestures and performs mapped task.
9	Yu et al. 2019 [51]	1 female elderly	Smart home for elders (SHfE) was developed to continuously monitor daily behaviors and living environments in their homes using unobtrusive sensors. Method: genetic algorithm, ant colony algorithm, and particle swarm optimization method.	Small and lightweight unobtrusive sensors to collect data of daily activities Data is collected every 30s and are fixated at specific locations in the house to collect data about temperature, water usage, electricity etc. Data collected is used to monitor the ADL of the elderly by caregivers.	SHfE is a good way to monitor daily activities and living environment.
10	Jekel et al. 2016 [52]	65-80 year old patients with MCI and healthy elderly	Smart home for MCI patients. A two-room flat equipped with video cameras and activity sensors Method: Kolmogorov-Smirnov-Z test, Kendall’s tau (τ), Chi-square tests	A two-room furnished flat with daily use objects like television, bookshelves, armchairs, telephone etc. ADL functioning was evaluated by giving a set of simple tasks like preparing a meal, using the telephone, fetching object from around the house etc.	Using technology, it was possible to evaluate and observe how patients performed task sand give an insight on the early signs of cognitive decline and helped in early detection on MCI.
11	Gnanavel et al. 2016 [53]	Proposed system, no subjects	Smart home monitoring system based on wireless sensor network for elderly for health monitoring and providing a secure living. Method: Fall detection algorithm.	A fall detection system comprised of a tri-axial accelerometer and gyroscope. Heartbeat sensor, pressure sensor and temperature sensor to monitor the health and wellness. 27SMS alert to the caregiver in case of any anomaly	The system is efficient and consumes less power and provide a safe and secure living to the elderly.
12	Rizvi et al. 2018 [54]	Proposed system for elderly/blind//handicapped people	Smart home system which can be controlled locally by Bluetooth and remotely by GSM technology 1Method: Android, Bluetooth technology.	This contains two systems: GSM module and Bluetooth module. GSM module can be used to connect remotely to appliances in the house via SMS or through specially designed android app. Bluetooth can be used to connect to appliances locally via the specially designed app in mobile phones	Targeted people’s life will improve and become easier. They will be able to carry out their ADL more efficiently and comfortably.
13	Lotfi et al. 2012 [55]	Elderly people with dementia Sample size: Not mentioned	Identification and prediction of anomalous behavior in elderly with dementia living in their homes by unobtrusive monitoring of activities of daily living Method: Echo State Network (ESN), Back Propagation Through Time (BPTT) and Real Time, Recurrent Learning (RTRL), RNN (Recurrent Neural Networks)	Data is collected and analyzed data is then transmitted to a secured central website for analysis by the caregiver/relative. The caregivers can remotely receive reports or alerts on a daily/weekly basis through e-mail or phone calls.	The caregiver is informed in case of detection of any anomalous behavior.
14	Nisar et al. 2016 [56]	Proposed system, no subjects	Information and communication technologies based smart home using android platform. Method: Android and Observe, Learn, and Adapt (OLA) algorithm using AI techniques.	Android based smart homes, the application has 3 main modules: sensor module, control module and actuator module. Sensors are fixed all over the house for instance door sensor, smoke detector, gas sensor, temperature sensor, humidity sensor. Devices can be monitored, and status can be controlled and accessed remotely as well	Efficient system prevents wastage of electricity and improves the quality of live for elderly.
15	Saunders et al. 2016 [57]	T&L component 20 subjects Interaction component 3 subjects	A commercially available robot has been set up in a sensor-based house Method: Decision trees/rule induction, HMM’s and dynamic Bayesian networks, k-NN dynamic windowing techniques	Teaching learning approach is used for the robot. Robot can be personalized to meet the changing needs.	Participants found the personalization of robots was easy and useful and can be used in real life by them.
16	Do et al. 2018 [58]	Graduate students Sample Size: 10	RiSH is able to evaluate auditory perception services, recognize activities of human body, track position, monitor human activities based on sound, detect fall and undertake rescue. Method: Dynamic Bayesan Network based Robot	The RiSH comprises of a robot for home service, a sensor network across the home, body sensors for monitoring activities, a mobile device, cloud-based servers, and remotely available caregivers.	37 distinct human activities were recognized by the robot through sound events with a 88% mean accuracy; falling sounds were detected with 80% accuracy at the frame level. The experiments showed the actions of modules in the RiSH and demonstrated the abilities of the home service robot in examining and aiding the resident.
17	Bennaser et al. 2019 [59]	Elderly people Sample size: Not mentioned	Environmental sensors-based platform was developed to monitor ADL including activities in kitchen, bathroom, living room, and bedroom. The behavior of older people was analyzed based on a set of ADLs. The platform comprised of many sensing modalities which are categorized into three: low-powered sensors worn on the body; sensors for environment; and sensors for power consumption. Method: Customized Machine Learning algorithms	The STRETCH platform is tri-layered system: a sensor-based network; centralized data analysis layer; and intervention layer. The collected data from the sensors is communicated to the central server securely through internet by the gateway. The communicated data is encrypted and is secured via a password.	STRETCH enables the data integration from sensors and their real-time transmission; it allows the sharing of ADL information.
18	Taramasco et al. 2019 [60]	Proposed system No subjects	Platform based tele-monitoring of ADL using non-intrusive sensors to take care of elderly; user-centered protocol guarantees the privacy of subjects. Method: Used AI modules SVM, LSTM, GRU and BLSTM	Installation of the system was done in the homes of the elderly: ◦ night-monitoring sensors ◦ noninvasive non-intrusive falling sensors ◦ humidity and carbon monoxide sensors ◦ Button to call his/her contacts in emergency. ◦ Connected with emergency and firefighter department. ◦ Early warning generation by platforms	Information of the patient’s situation at home and their ADL are sent in the form of daily, monthly, and annual reports according to data collected. Through data mining, advanced analysis was done to find significant patterns: characteristics of the disability, dependency of older age, among others, in order to prepare plans, programs, policies and treatments.
19	Iakovakis et al. 2016 [61]	15 Elderly (10 Parkinson’s disease and 5 healthy)	An assistive fall prevention tool based on fuzzy logic and accessible sensor-based technology, such as smartwatch, which aids in monitoring of risk factor for fall caused by orthostatic hypotension (OH) Method: Fuzzy logic	Crucial signal information collected from smartwatch and other motion-based sensors and the anticipated risk was calculated by using decision-making based on fuzzy logic.	This strategy makes way for complete utilization of the smartwatch data, such as HRV, to sustain QoL in activities of daily living.
20	Yu et al. 2015 [62]	5 Healthy elderly	A system was developed with ubiquitous sensors in the smart home Socialized Prompting System (SPS), which combines sensors and mobile based social networks to increase adherence to medicines Method: hierarchical agglomerative clustering algorithm for creating a community.	Smooth monitoring of medicine consumption behaviors was possible with the use of sensors, while community social prompting was done by the mobile social networks.	Use of the system improved medication adherence by the subjects.
21	Tsukiyama 2015 [63]	1 Healthy elderly	Evaluation of the health status of elderly living alone was done by a sensor-based monitoring system, based on their ADL. The system is able to predict and inform the healthcare center about any emergency situations without user interaction. Method: the reasoning program	Three activities of daily living were monitored: urination, kitchen work, and maintenance of physical hygiene. These activities are essential for a healthy lifestyle and are associated with tap water usage. Utilized water-flow sensors, infra-red-based motion sensors, and radio-frequency identification (RFID) receivers to screen the everyday life activity of an elderly and to identify any irregular situation.	The prototype was tested in a real home and it yielded anticipated results from the water flow based sensors.
22	Suryadevara et al. 2013 [64]	Healthy elderly Sample Size not mentioned	The ability to establish the well-being of an elderly living alone in a smart home using an economical, robust, flexible, and data-driven intelligent system. Method: Sensors Activity Pattern, Matching (SAPM) technique	The prototype is used for predicting the performance and wellness of the elderly by observing the daily usage of applications in a smart home. Acquiring the sensor data placed at various points and generating a model that can forecast the overall well-being of an elderly.	94% accuracy of activity recognition and estimating mechanism.
23	Grguric et al. 2019 [65]	Elderly people Sample size: Not mentioned	Utilizes concepts of AI like reasoning, pattern detection, decision making, and depends on Ambient Intelligence (AmI), Human-Computer Interaction (HCI) and sensors. Method: reasoning, pattern detection, decision making and cluster-based machine learning algorithms.	Non-visual sensor data and pattern recognition technique was powered by this data, was utilized based on low-cost, unobtrusive sensors. Distinctive behavioral patterns were learnt without compromising the privacy of the user. It warned caregiver(s) in case of detection of any abnormality.	A typical household can be easily converted to a smart household using this system.
24	Yu et al. 2012 [66]	15 Healthy Elderly	A fall detection system was developed based on computer vision to monitor the elderly in the home. Method: SVM	Digital video camera was used for vision-based method by transformation of the video frame by utilizing image processing techniques into features, using a Support Vector Machine (SVM) classifier to create a classification model.	97.08% high fall detection system rate was achieved; 0.8% false detection rate was seen in the simulated home.
25	Portet et al. 2013 [67]	Healthy elderly people Sample size: 8	User-friendly technology for automation of home on the basis of voice commands; evaluation of performance was done. Method: Voice command-based system.	A voice command-based interface was designed for the smart home. The performance of the system and its acceptance among elderly was evaluated.	The speech technology made everyday life easier. It can warn in case of dangerous situations and facilitate people to call for aid in case of a fall.
26	Hattink et al. 2016 [68]	42 elderly with MCI or dementia and 32 informal caregivers	Rosetta system was installed in the homes of patients with MCI or dementia in the experimental group which help in daily activities, recorded data and detected urgent situations to inform caregivers. Method: Application on Mobile Device.	Rosetta system consisted of subsystems: The Elderly day navigator The Early detection system The Unattended Autonomous Surveillance	Rosetta was accepted by users and they found it very useful.
27	Lupiani et al. 2015 [69]	25 Healthy elderly	Smart homes integrated with a Case Based Reasoning (CBR) approach for analyzing daily activities of elderly using diverse algorithms Method: t-CNN, t-RENN	Different types of activities were recognized (normal activity, bad night, etc.) for the elderly	The analyzed temporal CBM algorithms successfully reduced case-bases for detection of unusual scenarios.
28	Rudzicz et al. 2015 [70]	10 Elderly with Alzheimer’s disease	A mobile robot for assisting in daily living by monitoring visually and providing verbal prompts in cases of any issues for the elderly. Method: Machine learning with Robots	Speech-based interaction between 10 elderly human subjects and robots was studied to identify the issues or confusion that can pen.	The interaction during an ADL is challenging to detect and highly prone to a “lack of uptake,” which is the most common problem indicating verbal behavior among subjects
29	Hu et al. 2020 [71]	Proposed system, no subjects	Internet of Things technology to develop smart home care services and help to distribute instant information to remote users outside the domain Method: peer-to-peer (P2P) network using MQTT protocol.	A peer-to-peer (P2P) network system on which a camera array will help in identifying falling, transmitting alert events and supplying taken media streams in an elderly activity area. Multiple cameras with Wi-Fi and IoT functions arrange an inhouse P2P network where cameras can publish, subscribe and relay media content.	A practical scenario of elderly fall detection and alerting media sharing services in elder living environments is exhibited.
30	Bianchi et al. 2019 [72]	Proposed system for the elderly	HAR system, uses wearable devices integrated with the skills of deep learning techniques, to recognize the most popular daily activities of a person at home. Method: wearable device, CNN	The designed wearable sensor inserts an inertial measurement unit (IMU) and a Wi-Fi section to transmit data on a cloud service The sensor is linked to a convolutional neural network (CNN).	The system is created for daily activity supervision and nine different activities can be highlighted with an accuracy of 97%.
31	Fischinger et al. 2016 [73]	49 Healthy elderly	A robot named ‘Hobbit’ that assists the elderly living alone at home Method: Home-based robot with search-based planning (SBPL) algorithm for robot path planning	A care robot with capability of detecting and preventing falls and handling and detecting emergency. The interaction with the robot was done on a daily basis; the tasks included reminders, fetching objects, and entertaining. Recognizing speech automatically, text-to-speech conversion, recognition of gestures, and a graphical user interface based on touch.	The core task was executed very accurately by the robotic system. All tasks were performed by the elderly together with the robot and was evaluated as usable and acceptable.

**Table 2:** Features of smart home technology.

Study	Wearable	Body Sensors	Environmental Sensors	Camera	Voice Command	AI and ML	Robots	Privacy Preserving	Fall Detection	ADL Monitorirng	Feedback Provided by User
Kim et al. 2017 [43]	No	No	Yes	No	No	Yes	No	Yes	No	Yes	No
Deen 2015 [44]	Yes	Yes	Yes	No	No	Yes	No	Yes	Yes	Yes	No
Sprint et al. 2016 [45]	No	No	Yes	No	No	Yes	No	No	Yes	Yes	No
Wilson et al. 2019 [46]	No	No	Yes	Yes	No	Yes	Yes	Not mentioned	Yes	Yes	Yes
Alberdi et al. 2018 [47]	No	No	Yes	No	No	Yes	No	Yes	No	Yes	No
Dawadi et al. 2013 [48]	No	No	Yes	No	No	Yes	No	Not mentioned	No	Yes	No
Aramendi et al. 2018 [49]	No	No	Yes	No	No	Yes	No	Yes	No	Yes	No
Kshirsagar et al. 2020 [50]	Yes	Yes	Yes	No	No	Yes	No	Not mentioned	No	No	Yes
Yu et al. 2019 [51]	No	No	Yes	No	No	No	No	Yes	No	yes	Yes
Jekel et al. 2016 [52]	No	No	Yes	Yes	No	No	No	No	No	Yea	Yes
Gnanavel et al. 2016 [53]	No	Yes	Yes	No	No	No	No	Not mentioned	Yes	Yes	No
Rizvi et al. 2018 [54]	No	No	Yes	No	No	No	No	No	No	No	No
Lotfi et al. 2012 [55]	No	No	Yes	No	No	Yes	No	Yes	No	Yes	No
Nisar et al. 2016 [56]	No	No	Yes	No	No	No	No	No	No	Yes	No
Saunders et al. 2016 [57]	No	No	Yes	No	Yes	Yes	Yes	Not mentioned	No	No	Yes
Do et al. 2018 [58]	Yes	No	Yes	Yes (on robot)	No	Yes	Yes	Yes	Yes	Yes	No
Bennaser et al. 2019 [59]	Yes	Yes	Yes	No	No	Yes	No	Yes	No	Yes	No
Taramasco et al. 2019 [60]	No	No	Yes	Yes	No	Yes	No	Yes	Yes	No	No
Iakovakis et al. 2016 [61]	Yes	Yes	Yes	No	No	Yes	No	Not Mentioned	Yes	Yes	No
Yu et al. 2015 [62]	No	No	Yes	No	No	Yes	No	No	No	No	No
Tsukiyama 2015 [63]	No	No	Yes	No	No	Yes	No	Not mentioned	No	Yes	No
Suryadevara et al. 2013 [64]	No	No	Yes	No	No	No	No	No	No	Yes	No
Grguric et al. 2019 [65]	No	No	Yes	No	No	Yes	No	Yes	No	No	Yes
Yu et al. 2012 [66]	No	No	No	Yes	No	Yes	No	Not Mentioned	Yes	Yes	No
Portet et al. 2013 [67]	No	No	Yes	Yes (video conference)	Yes	No	No	Yes	Yes	Yes	Yes
Hattink et al. 2016 [68]	No	No	Yes	Yes	No	No	No	No	No	No	NO
Lupiani et al. 2015 [69]	No	No	Yes	No	No	Yes	NO	Not mentioned	No	Yes	No
Rudzicz et al. 2015 [70]	No	No	No	Yes (on robot)	Yes	Yes	Yes	Not mentioned	No	No	Yes
Rudzicz et al. 2015 [70]	No	No	No	Yes (on robot)	Yes	Yes	Yes	Not mentioned	No	No	Yes
Hu et al. 2020 [71]	No	No	Yes	Yes	No	No	No	No	No	no	No
Bianchi et al. 2019 [72]	Yes	Yes	No	No	No	Yes	No	Not mentioned	No	No	No
Fischinger et al. 2016 [73]	No	No	No	No	Yes	Yes	Yes	Not mentioned	No	No	Yes

## Abbreviations

SVM	Support Vector Machine
RiSH	A robot-integrated smart home
STRETCH	Socio-Technical Resilience for Enhancing Targeted Community Healthcare
ADL	Activities of Daily Living
LSTM	Long-Short Term Memory
GRU	Gated Recurrent Units
BLSTM	Bidirectional Long Short-Term Memory
CASAS-AR	Center for Advanced Studies in Adaptive Systems-Activity Recognition
ML	Machine Learning
GSM	Global System for Mobile Communications
HMM	Hidden Markov Model
MQTT	Message Queuing Telemetry Transport
